# Protein kinase D3 modulates MMP1 and MMP13 expression in human chondrocytes

**DOI:** 10.1371/journal.pone.0195864

**Published:** 2018-04-13

**Authors:** Jonathan Baker, Adrian M. D. Falconer, David J. Wilkinson, G. Nicholas Europe-Finner, Gary J. Litherland, Andrew D. Rowan

**Affiliations:** Skeletal Research Group, Institute of Genetic Medicine, Newcastle University, Newcastle-upon-Tyne, United Kingdom; Dasman Diabetes Institute, KUWAIT

## Abstract

Many catabolic stimuli, including interleukin-1 (IL-1) in combination with oncostatin M (OSM), promote cartilage breakdown via the induction of collagen-degrading collagenases such as matrix metalloproteinase 1 (*MMP1*) and *MMP13* in human articular chondrocytes. Indeed, joint diseases with an inflammatory component are characterised by excessive extracellular matrix (ECM) catabolism. Importantly, protein kinase C (PKC) signalling has a primary role in cytokine-induced *MMP1/13* expression, and is known to regulate cellular functions associated with pathologies involving ECM remodelling. At present, substrates downstream of PKC remain undefined. Herein, we show that both IL-1- and OSM-induced phosphorylation of protein kinase D (PKD) in human chondrocytes is strongly associated with signalling via the atypical PKCι isoform. Consequently, inhibiting PKD activation with a pan-PKD inhibitor significantly reduced the expression of *MMP1/13*. Specific gene silencing of the PKD isoforms revealed that only PKD3 (*PRKD3*) depletion mirrored the observed *MMP* repression, indicative of the pharmacological inhibitor specifically affecting only this isoform. *PRKD3* silencing was also shown to reduce serine phosphorylation of signal transducer and activator of transcription 3 (STAT3) as well as phosphorylation of all three mitogen-activated protein kinase groups. This altered signalling following *PRKD3* silencing led to a significant reduction in the expression of the activator protein-1 (AP-1) genes *FOS* and *JUN*, critical for the induction of many MMPs including *MMP1/13*. Furthermore, the AP-1 factor activating transcription factor 3 (*ATF3*) was also reduced concomitant with the observed reduction in *MMP13* expression. Taken together, we highlight an important role for PKD3 in the pro-inflammatory signalling that promotes cartilage destruction.

## Introduction

The progression of joint diseases such as rheumatoid arthritis (RA) and osteoarthritis (OA) is associated with inflammation, whereby inflammatory mediators released by infiltrating immune cells as well as resident joint cells induce altered gene expression that promotes extracellular matrix (ECM) degradation [1]. Cytokines such as interleukin (IL-)1 and tumour necrosis factor α (TNFα) are key mediators in the inflammatory responses in destructive joint disease [2,3], and we have previously demonstrated enhanced cartilage ECM catabolism by these mediators when combined with IL-6-family cytokines such as oncostatin M (OSM) *in vitro* and *in vivo* [4–8]. This co-operative exacerbation in catabolic potential is likely to occur in many inflammatory milieu, such as the arthritides, to promote cartilage ECM catabolism.

Normal articular cartilage is maintained by a single resident cell type, the chondrocyte, which preserve homeostasis by regulating the expression of ECM components and catabolic factors such as the matrix metalloproteinases (MMPs). Collectively, the MMPs can degrade all ECM macromolecules, and during inflammatory joint diseases stimulated chondrocytes secrete elevated levels of MMPs which, once activated, mediate proteolysis of tendon, bone and cartilage [9,10]. MMP-1 and MMP-13 are the collagenolytic MMPs most strongly associated with cartilage collagenolysis, a key proteolytic event in joint diseases since it is essentially irreversible [11]. Indeed, MMP-13 is the most potent collagenase with respect to type II collagen [12], the major structural collagen in articular cartilage. Potent pro-inflammatory stimuli such as IL-1+OSM activate a complex array of signal transduction pathways, shown to be common to a wide variety of pro-inflammatory stimuli [13], which together markedly enhance the induction of *MMP1/13* in human chondrocytes [7,13–15]. The cFos/cJun activator protein-1 (AP-1) transcription factor complex is critical for *MMP* gene expression (see [16] and references therein), although we recently reported a role for the AP-1-binding factor activating transcription factor 3 (ATF3) in selectively mediating *MMP13* expression which was nevertheless AP-1-dependent [13]. Furthermore, we have also previously demonstrated the importance of signal transducer and activator of transcription (STAT)3, phosphatidylinositol-3’ kinase (PI3K/Akt) and protein kinase C (PKC) signalling pathways [17–19]. The atypical PKC isoform PKCι has been shown to modulate *MMP1/13* induction following IL-1+OSM stimulation [19], but the identity of the downstream signalling components it affects remains unknown, as do potential points of cross-talk.

Protein kinase D (PKD) comprises a family of serine/threonine protein kinases that belong to the Ca^2+^/calmodulin-dependent kinase superfamily. Three isoforms exist in humans: PKD1, PKD2 and PKD3 (reviewed in [20]). Diverse cellular functions, including cell survival/proliferation and apoptosis, plasma membrane-directed transport, metastasis and inflammation have been reported [20–24] for PKD isoforms. With respect to cartilage remodelling, PKD is reported to regulate both the expression and activity of several MMPs. Studies have shown that PKD1 down-regulates the expression and activity of multiple MMPs in breast cancer cells lines, particularly the gelatinase MMP-9 [22]. Conversely, gene silencing of PKD2 and PKD3 leads to a decrease in the activity of MMP-9 in prostate cancer, suggesting MMP gene regulation by distinct PKD family members is cell-type specific. PKD3 has been reported to regulate nuclear localization and activity of histone deacetylases 5 and 7 (HDAC5/7) [25]. OA cartilage exhibits elevated HDAC7 levels, whilst *HDAC7* depletion in SW1353 human chondrosarcoma cells strongly suppressed IL-1-dependent induction of *MMP13* [26]. PKD3 can be activated independently of PKC via phosphorylation of tyrosine residues within the pleckstrin homology domain following oxidative stress, which is a feature of OA [27]. Moreover, PKD isoforms have also been shown to be a downstream target of G-protein-coupled receptors, via increases in phospholipase C activity and auto-phosphorylation [28]. Such receptors play roles in innate and adaptive immunity, and have been implicated in the pathology associated with arthritis [29–31].

PKD3 is ubiquitously expressed in adult human tissues [32] and has been shown to be expressed during skeletogenesis in mice, notably in the cartilage primordia of bones [33], which are areas of active ECM turnover (via MMP-13) as cartilage is remodelled prior to mineralization. Interestingly, phenotypic analysis of a mutant mouse strain harbouring a gene-trap deletion of the PKD3 gene (*prkd3*) revealed a mild skeletal abnormality including decreased mean trabecular bone volume and thickness [34], suggestive of reduced remodelling in cartilage primordia. In this context, downstream PKD3 signalling may also be involved in cytokine-induced *MMP13* expression in human chondrocytes. Herein, we show that PKD3 is indeed involved in pro-inflammatory signalling via modulating the expression of the AP-1 genes *FOS* and *JUN*, and enhancing *MMP* expression. PKD3 activity also stimulates the induction of *ATF3* which is specifically involved in cytokine-induced *MMP13* expression in human chondrocytes.

## Materials and methods

### Materials

All chemicals were obtained from Sigma Chemical Co (Poole, UK) unless otherwise stated and of the highest purity available. All cytokines were recombinant human. IL-1α was a generous gift from Dr Keith Ray (GlaxoSmithKline, Stevenage, UK). OSM was prepared in-house using expression vectors kindly provided by Prof. J. Heath (University of Birmingham, UK) and methods described [35]. Gö6983 was from Merck Chemicals (Nottingham, UK). PKD inhibitor kb NB 142–70 was from Tocris Bioscience (Bristol, UK). The STAT3 inhibitor S31-201 was from Selleckchem (TX, USA). Kinase inhibitors and small interfering (siRNA) reagents were screened for toxicity using the Toxilight assay of adenylate kinase release (Lonza, Wokingham, UK), and always used at concentrations that did not affect cell viability over the assay period.

### Chondrocytes

Human chondrocytes were obtained by the enzymatic digestion of macroscopically normal articular cartilage from OA patients undergoing joint replacement surgery as described [36]. All subjects gave informed consent and the study was approved by the Newcastle and North Tyneside Joint Ethics Committee (REC 14/NE/1212). Chondrocytes were maintained in Dulbecco’s modified Eagle’s medium (DMEM) supplemented with 10% foetal bovine serum (FBS), 100 IU penicillin, 100 μg/ml streptomycin, 40 U/ml nystatin.

### Cell fractionation and immunoblotting

Chondrocyte lysates were prepared as described previously (18) and stimulated with IL-1 (0.2 ng/ml) ± OSM (10 ng/ml) for up to 60 min. In some experiments, chondrocytes were subjected to subcellular fractionation using NE-PER Nuclear and Cytoplasmic Protein Extraction Kit or Subcellular Protein Fractionation Kit (both from ThermoFisher Scientific, Loughborough, UK). GAPDH was used as a loading control for whole cell lysates whilst MEK2 and lamin A/C were used for cytoplasmic and nuclear extracts, respectively. Lysates or fractions were resolved by SDS-PAGE, transferred to PVDF membranes and subsequently probed using the following antibodies: PKCι (#2998), PKCζ (#9372), PKD^*S916^ (#2051), PKD^*S744/748^ (#2054: recognises PKD^*S731/735^ in PKD3), PKD3 (#5655), ERK1/2^*T202/Y204^ (#9101), JNK (#9252), JNK^*T183/Y185^ (#9251), p38^*T180/Y182^ (#9211), Akt^*S473^ (#4060), cFos (#4384), STAT1^*Y701^ (#9171), STAT1^*S727^ (#9177), STAT3^*Y705^ (#9131), STAT3^*S727^ (#9134) and lamin A/C (#2032) were from Cell Signaling Technology (Danvers, MA); cJun (sc1694) and ATF3 (sc188) were from Santa Cruz Biotechnology (Santa Cruz, CA); glyceraldehyde 3’-phosphate dehydrogenase (GAPDH; MAB374) was from Millipore (MA, USA); PKD1 (L905) was from Bio-world (Dublin, USA); MEK2 (#04–377) and PKD2 (#1969–1) were from Epitomics (Insight Biotech, Wembley, UK). The specificity of all antibodies was confirmed using chondrocyte lysates (see full-length blots in S1 Fig), whilst blots were cropped for clarity of comparison.

### Gene silencing

*PRKC*Z and *PRKCI* siRNAs were Dharmacon ON-TARGETplus™ (ThermoFisher Scientific), comprising 4 specific siRNA complexes [16], whilst validated MISSION™ siRNA reagents (Sigma) were used to silence: *PRKD2*, 5’-CGAUACAUCACGCAUGAGA-3’; *PRKD3*, 5’-CAUAAACGCUGUGCAUCAA-3’; *JNK1*, 5’-GUUCUUAUGAAAUGUGUUATT-3’; *JNK2*, 5’-CUGUAACUGUUGAGAUGUATT-3’. Primary human chondrocytes were prepared and cultured as above, and transfected as described previously (18). After transfection, cells were serum-starved for 24 h prior to stimulation as indicated. Depletion of gene-specific mRNA levels was calculated by comparison of expression levels with cells transfected with 100 nM siCONTROL (siCon: non-targeting siRNA #2, Cat. 001210–02; Dharmacon).

GIPZ lentiviral vector containing non-targeting control shRNA (shCon) or *PRKD1* shRNA (sh*PRKD1*: V2LHS-170466) were purchased from OpenBiosystems (Thermo Fisher Scientific). Packaging and envelope plasmids pCMV-dR8.91 and pMD2.G were a kind gift from Prof. Nick Reynolds (Newcastle University). The HEK293T producer cell line (SBI, California, USA), maintained in DMEM plus 10% FBS, was employed for packaging. On the day of transfection, medium was changed to DMEM containing 10% heat-inactivated FBS. Packaging, envelope and shRNA plasmids were co-transfected using JetPEI transfection reagent (Polyplus, Illkirch, France), according to the manufacturer’s instructions. Supernatant was harvested 72 h post-transfection and filtered (0.45 μm). Viral particles were then concentrated 50-fold using a Lenti-X concentrator (Takara Bio Europe/ Clontech, Saint-Germain-en-Laye, France), and viral titre calculated using the Lenti-X qRT-PCR Titration Kit (Takara). Multiplicity of infection (MOI) was then calculated via serial dilution. Primary chondrocytes were prepared and cultured as above, and exposed to lentiviral infection at a MOI of 30 for 72 h. Transduction was assessed using confocal microscopy for green fluorescent protein (GFP). After transduction, cells were serum-starved for 24 h then stimulated as indicated.

### Real-time PCR of relative mRNA levels

Primary human chondrocytes were stimulated with IL-1 (0.05 ng/ml) ± OSM (10 ng/ml) for 1 h to measure *FOS*, *JUN* and *ATF3*, or 24 h for *MMP* mRNAs. RNA was stabilised in cell lysates in a 96-well format, cDNA synthesised and real-time PCR assays conducted using conditions described previously [18]. Primer and probe sequences are as previously detailed [13,19], or *MMP8*: For, 5’- CACTCCCTCAAGATGACATCGA-3’ and Rev, 5’-ACGGAGTGTGGTGATAGCATCA-3’, Probe, 5’-CAAGCAACCCTATCCAA CCTACTGGACCAA-3’; *MMP14*: For, 5’-AGGCCGACATCATGATCTTCTTT-3’ and Rev, 5’-AAGTGGGTGTCTCCTCCAATGTT-3’, Probe, 5’-CCATGGCGACAGCACGC CCTT-3’. Some Taqman assays used Universal Probe Library probes (Roche Applied Sciences) as directed: *PRKD1*: For, 5’- TGTATTACCCTCTTTCAGAATGACA-3’ and Rev, 5’-CCAGAGACAAAATTTCAGATAAAGG-3’, Probe: 38; *PRKD2*: For, 5’-AGATGCTC TTCGGCCTAGTG-3’ and Rev, 5’-AGCGCTTGTGGTAGTTCAGC-3’, Probe: 46; *PRKD3*: For, 5’-TGATTTAAAGCCAGAAAATGTGC-3’ and Rev, 5’-CGTGCAAATCC AAAGTCACA-3’, Probe: 21.

### Statistical analyses

Statistical differences between sample groups were assessed using one-way analysis of variance (ANOVA) with a post-hoc Bonferroni’s multiple comparison test or Student’s 2-tailed unpaired *t*-test (1 sample *t*-test for immunoblot quantification), where ***p<0.001, **p<0.01, *p<0.05. For clarity, only selected comparisons are presented in some Figures.

## Results

### Inhibition of PKD phosphorylation curtails IL-1+OSM induction of *MMP1* and *MMP13* in human articular chondrocytes

**Fig 1:**
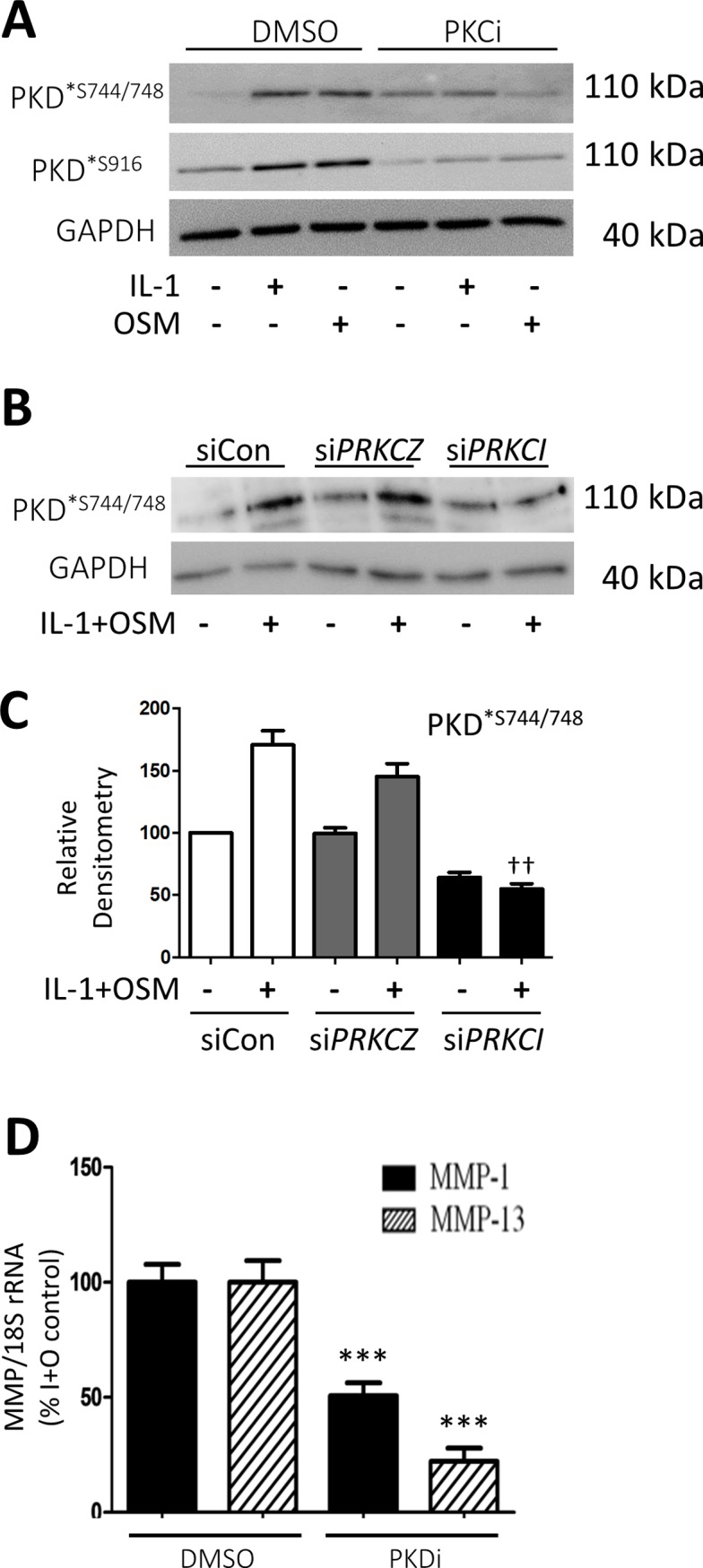
Effect of PKD inhibition on collagenase expression in human chondrocytes. Primary human articular chondrocytes were stimulated with IL-1 (0.2 ng/ml) alone, OSM (10 ng/ml) alone or IL-1+OSM as detailed in the Methods for 20 min (A-C). Cells were either (A) pre-treated with Gӧ6983 (20 μM; PKCi) for 1 h or (B and C) transfected with siRNA for non-targeting siCon (white bars), *PRKCZ* (grey bars) or *PRKCI* (black bars) (all 100 nM) for 48 h prior to stimulation. Cell lysates were prepared as described in Methods and subjected to SDS-PAGE and immunoblotting (using the antibodies indicated). Relative densitometry of some blots was performed, scans combined and plotted graphically (C), where ^††^, p≤0.01 versus siCon-treated IL-1+OSM. (D) Human chondrocytes were pre-treated with NB142-70 (5 μM; PKDi), or a DMSO vehicle control for 1 h prior to stimulation with IL-1 (0.05 ng/ml) + OSM (10 ng/ml) for 24 h. Real-time RT-PCR was performed on isolated RNA to determine relative levels of *MMP1* (black bars) and *MMP13* (hatched bars), normalised to *18S rRNA* housekeeping gene and presented as a percentage of the IL-1+OSM induction (n = 6; mean ± S.E.), where ***, p≤0.001 versus the relevant DMSO-treated vehicle control. Data are representative of at least 3 separate chondrocyte populations.

We previously demonstrated roles for PKCι and PKCζ in IL-1+OSM-mediated expression of the key collagenases *MMP1* and *MMP13* in human chondrocytes [19]. Moreover, we have also shown both IL-1 and OSM induce PKD phosphorylation such that we sought to confirm that PKD was indeed a downstream substrate of PKC signalling. Cytokine-induced PKD phosphorylation was assessed in the presence of the Gӧ6983 (20 μM), a PKC inhibitor that does not inhibit PKD, which completely abolished both IL-1- and OSM-stimulated PKD phosphorylation (Figs 1A and S2). Assessment of the effect of gene silencing of *PRKCI* and *PRKCZ* (coding for PKCι and PKCζ, respectively; S3A Fig) indicated that, of the atypical PKC isoforms, only *PKCι* played a role in this phosphorylation (Fig 1B and 1C).

Since combined PKC/PKD inhibition (using Gӧ6976) suppressed *MMP* expression and reduced cytokine-induced collagen release from cartilage [16], we confirmed that a specific PKD inhibitor (NB142-70; PKDi) also significantly reduced IL-1+OSM-induced expression of *MMP1* and *MMP13* expression (Fig 1D). Together, these findings implicate a role for PKD in cytokine-induced *MMP* expression in human chondrocytes.

### PKD3 depletion correlates with reduced *MMP1* and *MMP13* expression in human chondrocytes

**Fig 2:**
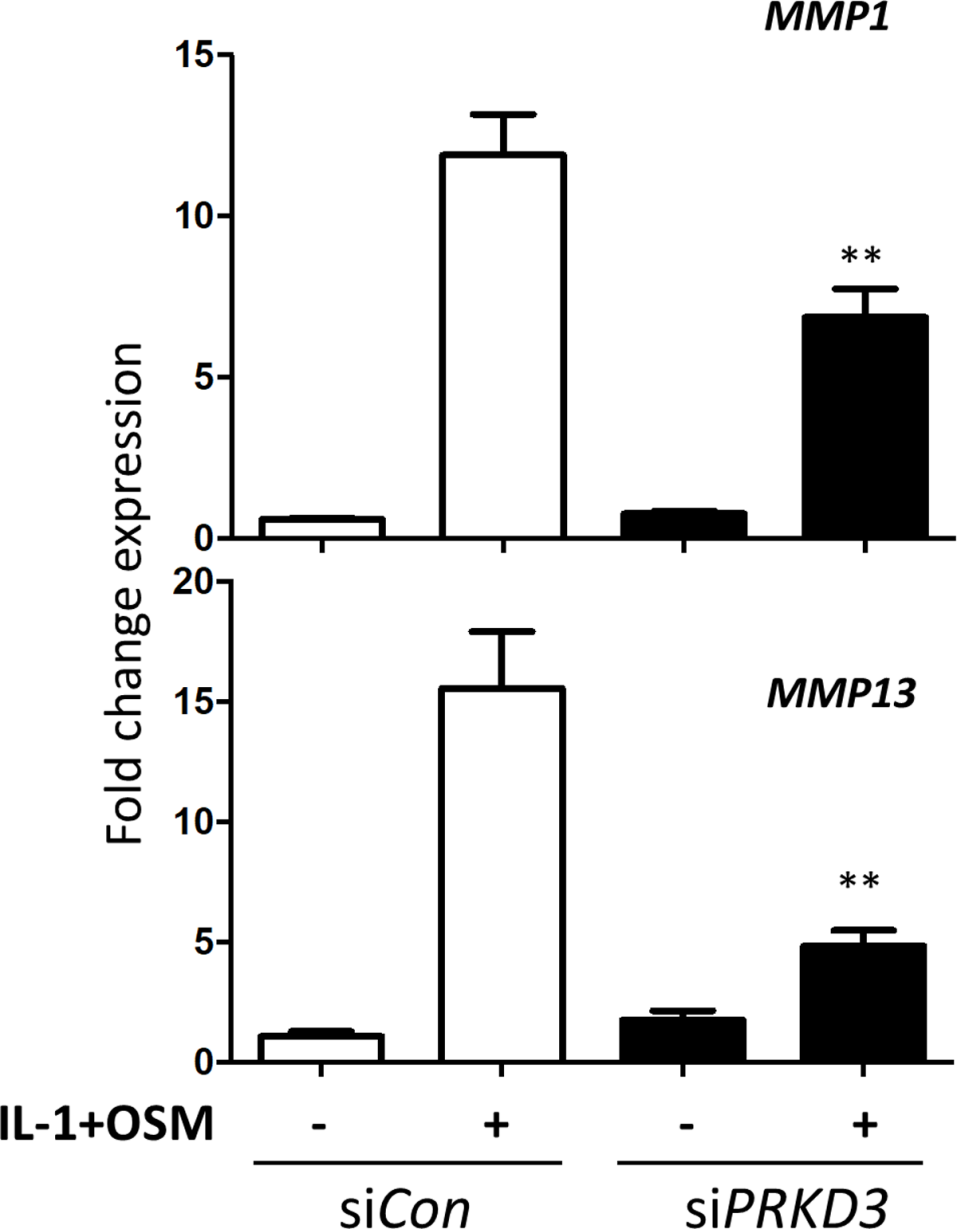
Effect of PKD isoform silencing on *MMP1* and *MMP13* expression in human chondrocytes. Following transfection with siRNA, either non-targeting siCon (white bars) or one specific for *PRKD3* (black bars) (both 100 nM), primary human articular chondrocytes were stimulated with IL-1 (0.05 ng/ml) + OSM (10 ng/ml) for 24 h. Cells were lysed and real-time RT-PCR performed for *MMP1* (*upper panel*) and *MMP13* (*lower panel*), 72 h after the start of transfection as described in the Materials and Methods. Data (mean ± S.E.) are representative of at least three separate chondrocyte populations (each assayed in hextuplicate), presented as relative expression levels normalised to *18S rRNA* housekeeping gene, where **, p≤0.01 versus siCon transfection.

To determine which of the three individual PKD isoforms accounted for the PKDi-mediated reduction in *MMP* expression, selective ‘knockdown’ of the PKD isoforms (S3B Fig) indicated that only *PRKD3* silencing led to a significant decrease in the expression of both *MMP1* and *MMP13* (Fig 2). Expression of these MMPs was markedly enhanced with *PRKD1* silencing but unaltered following *PRKD2* depletion (S4A and S4B Figs, respectively). Since these data strongly implicated PKD3 as the only ‘pro-inflammatory’ moiety of PKD signalling for cytokine-induced *MMP* expression, specificity assessment of *PRKD3* silencing for the other collagenolytic MMP species indicated that *MMP8*, but not *MMP14*, was also significantly suppressed (S5 Fig).

### PKD3 depletion alters STAT signalling in IL-1+OSM-stimulated human chondrocytes

**Fig 3:**
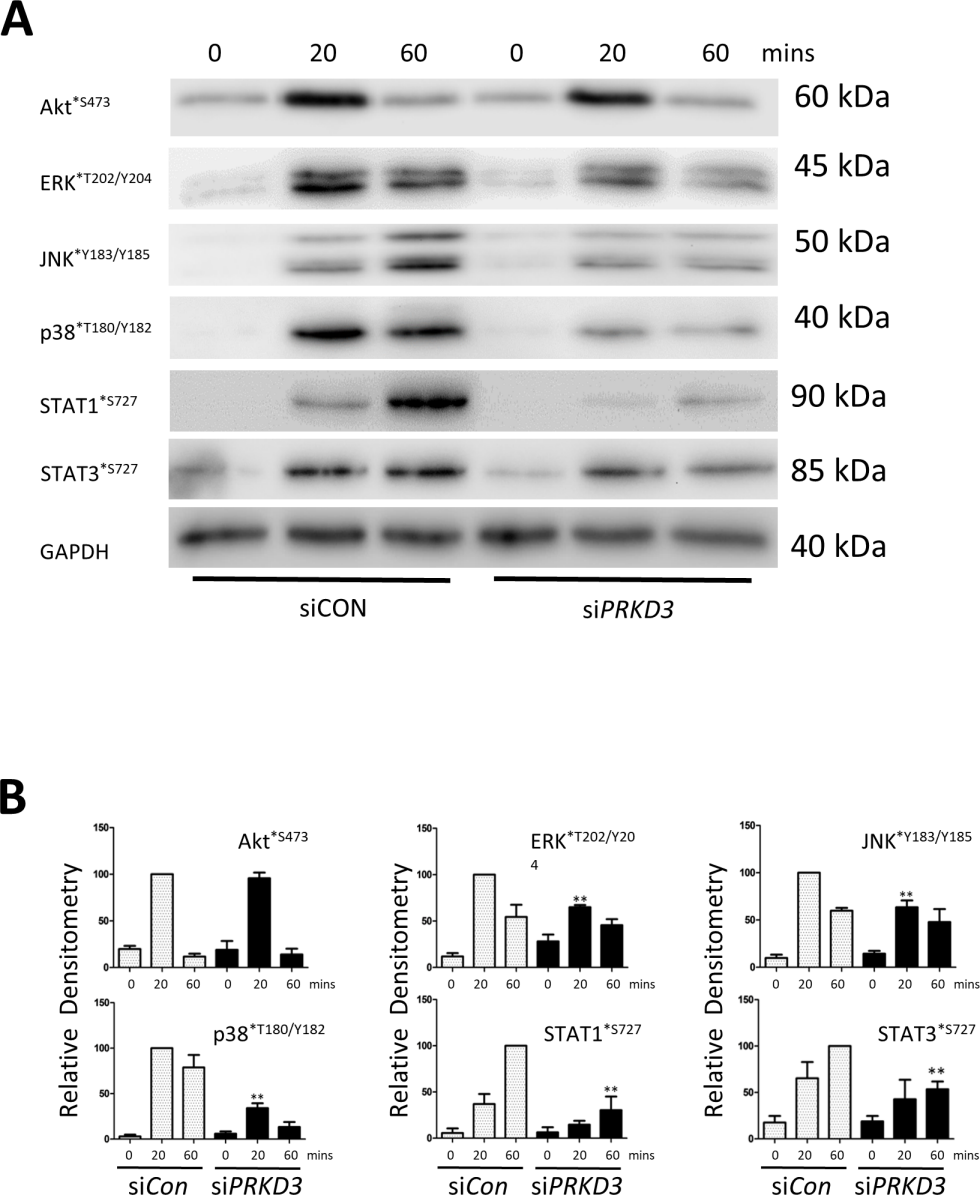
Effect of *PRKD3* silencing on STAT and MAPK phosphorylation in human chondrocytes. (A) Following transfection with siRNA specific to *PRKD3* or non-targeting siCon (100 nM), primary human articular chondrocytes were stimulated with IL-1+OSM as detailed in the Methods for the indicated times. Whole cell lysates were prepared and subjected to SDS-PAGE and immunoblotting using the antibodies indicated. (B) Scans from multiple blots with siCon (white bars) or si*PRKD3* (black bars) were combined and the relative densitometry values presented for the individual antibodies used, where **, p≤0.01 vs the relevant siCon time point. The data are representative of at least three separate chondrocyte populations.

**Fig 4:**
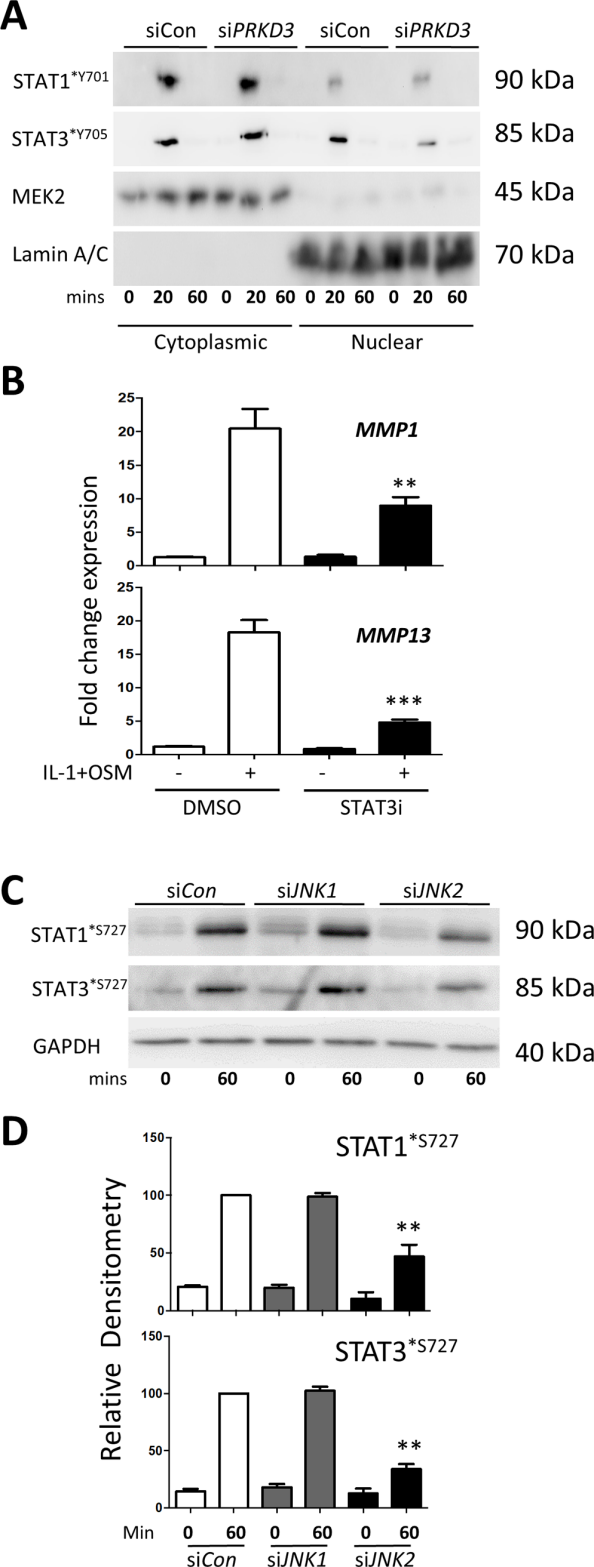
The role of STATs in PKD3-mediated *MMP* expression in human chondrocytes. (A) Following transfection with siRNA specific to *PRKD3* or non-targeting siCon (100 nM), primary human articular chondrocytes were stimulated with IL-1+OSM as detailed in the Methods for the indicated times. Subcellular fractions were prepared and subjected to SDS-PAGE and immunoblotting using the antibodies indicated. (B) Chondrocytes were pre-treated for 1 h with S31-201 (STAT3i; black bars) or DMSO vehicle control (white bars), and then stimulated with IL-1+OSM for 24 h. Lysates were subjected to real-time RT-PCR (n = 6; mean ± S.E.) for *MMP1* and *MMP13* as described in Methods. Data are presented as relative expression levels normalised to *18S rRNA* housekeeping gene, where ***, p≤0.001, *, p≤0.05 vs the relevant DMSO treatment. (C) Chondrocytes were transfected with siCon (white bars), si*JNK1* (grey bars) or si*JNK2* (black bars) (all 100 nM) for 48 h prior to stimulation with IL-1+OSM for the indicated durations. Cell lysates were immunoblotted with the antibodies indicated. Scans from multiple blots were combined and the relative density values presented for the individual antibodies used (D), where **, p≤0.01 vs the relevant siCon time point. All data are representative of at least three separate chondrocyte populations.

Analyses of the signalling pathways activated in human chondrocytes following *PRKD3* silencing with IL-1+OSM stimulation revealed reductions in phosphorylation of all three MAPK groups but no apparent effect on the phosphorylation of Akt^*S473^ (Fig 3A and 3B). As dual phosphorylation of STATs occurs for full activity [37,38], we also observed reductions in Ser^727^ phosphorylation of STAT1 and STAT3 (Fig 3A and 3B), important for transcriptional activity [37], whilst a more detailed analysis using subcellular fractionation indicated that although cytoplasmic (and indeed total) levels of Tyr^705^ phosphorylation of STAT3 appeared to be relatively unaffected, reduced nuclear levels were observed (Fig 4A) following *PRKD3* depletion. In contrast, total and nuclear STAT1^*Y701^ was unaffected by *PRKD3* silencing (Fig 4A).

To further confirm a role for STAT3 in IL-1+OSM-mediated *MMP* expression [17,19] the STAT3 inhibitor S31-201 significantly reduced both *MMP1* and *MMP13* expression in human chondrocytes (Fig 4B). Since *PRKD3* silencing curtailed IL-1+OSM-mediated JNK activation, and in light of the previously reported PKD-dependent activation of JNK [39] and our previous report indicating no dependency for ERK [19], we determined whether the observed effects on STAT phosphorylation could be mediated by JNK moieties. Gene silencing of *JNK1* and *JNK2* revealed that JNK2 appeared to have a role in the Ser^727^ phosphorylation of both STAT1 and STAT3 following IL-1+OSM stimulation (Fig 4C and 4D).

### PKD3 indirectly regulates AP-1 factors in IL-1+OSM-mediated *MMP13* expression

**Fig 5:**
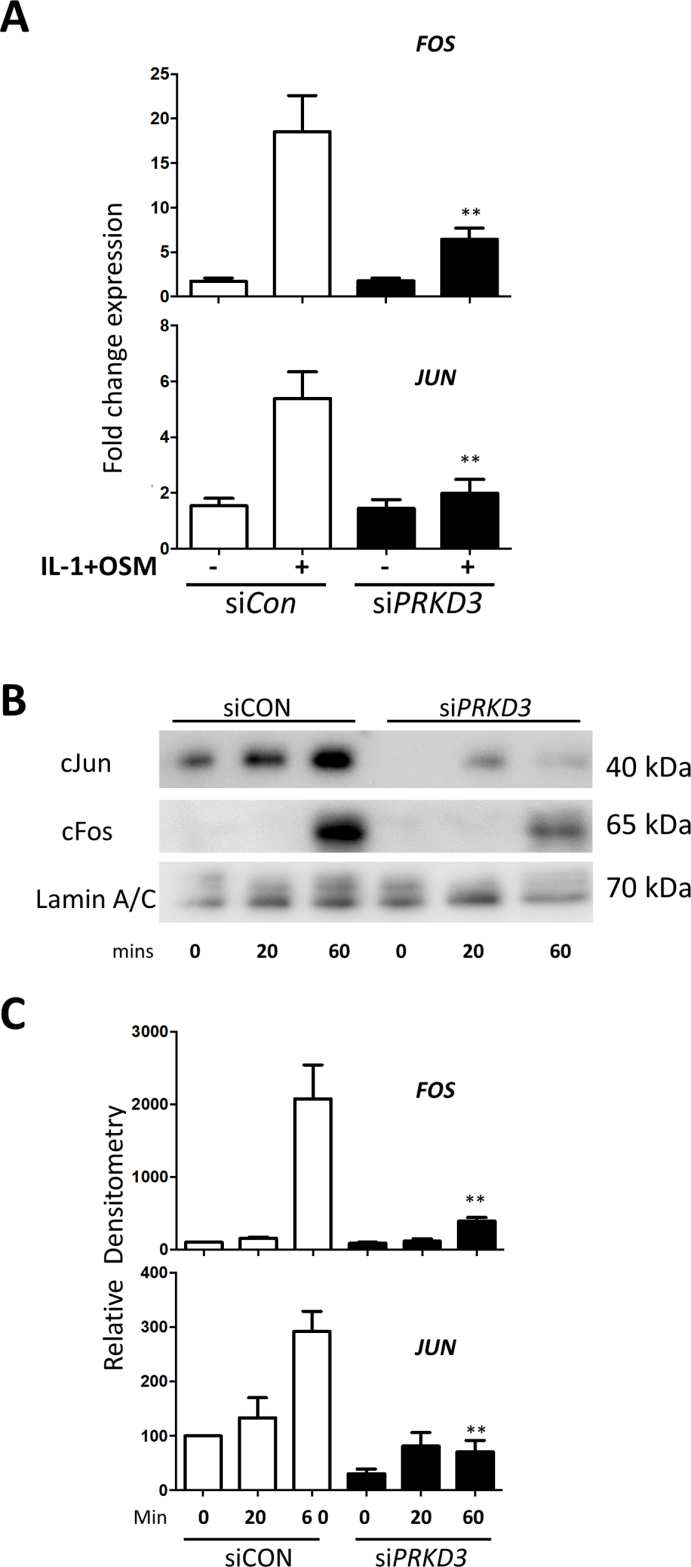
Effect of *PRKD3* gene silencing on the expression of AP-1 factors in human chondrocytes. Following transfection with siRNA specific to *PRKD3* (black bars) or non-targeting siCon (white bars) (both 100 nM), primary human articular chondrocytes were stimulated with IL-1+OSM as detailed in the Methods for 1 h unless stated otherwise. (A) Cell lysates were subjected to real-time RT-PCR (n = 6; mean ± S.E.) for *FOS* (*upper panel*) or *JUN* (*lower panel*) as described in Methods. Data are presented as relative expression levels normalised to *18S rRNA* housekeeping gene, where **, p≤0.01 versus siCon. (B and C) Nuclear fractions were prepared and subjected to SDS-PAGE and immunoblotting using the antibodies indicated. Scans from multiple blots were combined and the relative density values presented for the individual antibodies used (C), where **, p≤0.01 vs the relevant siCon time point. All data are representative of at least three separate chondrocyte populations.

**Fig 6:**
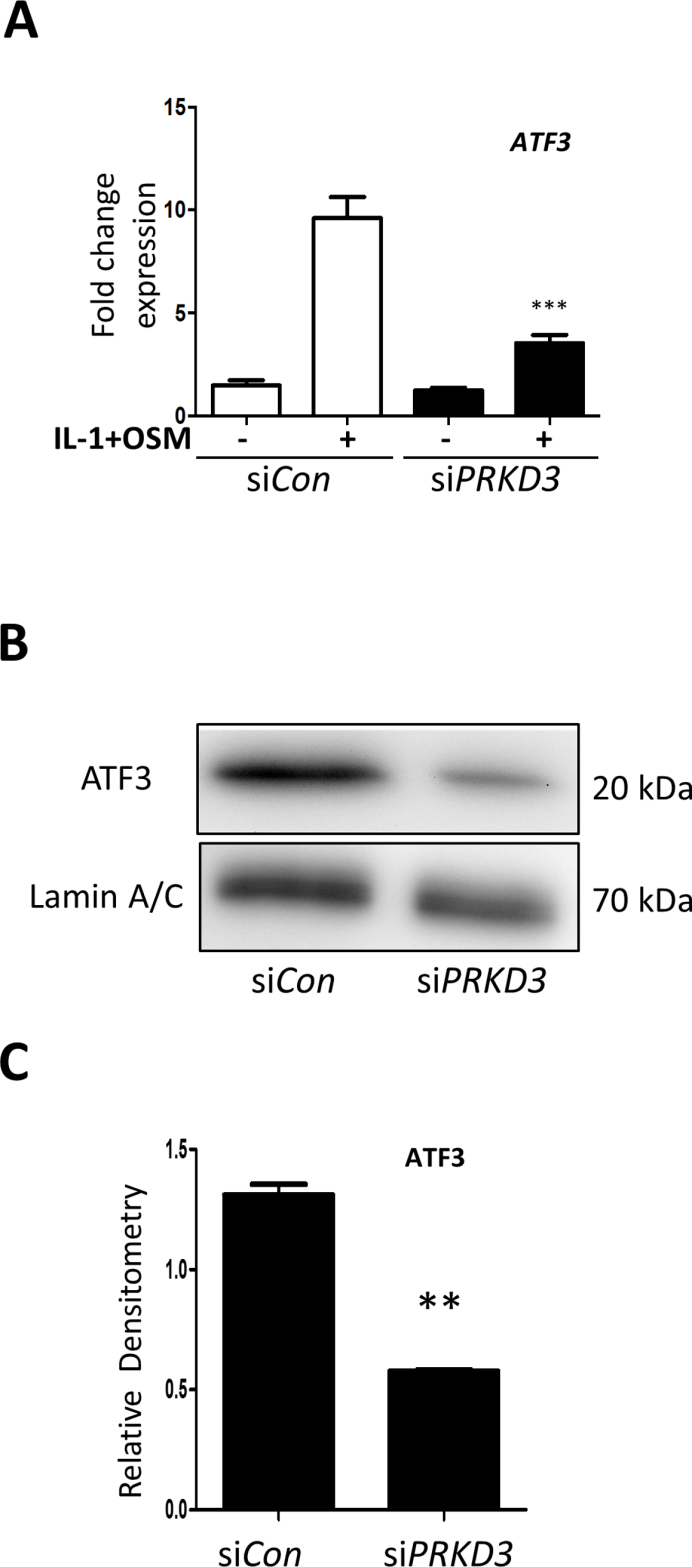
Effect of *PRKD3* gene silencing on *ATF3* expression in human chondrocytes. Following transfection with siRNA specific to *PRKD3* (black bars) or non-targeting siCon (white bars) (both 100 nM), primary human articular chondrocytes were stimulated with IL-1+OSM as detailed in the Methods for 75 min unless stated otherwise. (A) Cell lysates were subjected to real-time RT-PCR (n = 6; mean ± S.E.) for *ATF3* as described in Methods. Data are presented as relative expression levels normalised to *18S rRNA* housekeeping gene, where ***, p≤0.001 versus siCon. (B) Nuclear fractions were prepared and subjected to SDS-PAGE and immunoblotting using the antibodies indicated. Scans from multiple blots were combined and the relative density values presented (C), where **, p≤0.01 vs siCon. All data are representative of at least two separate chondrocyte populations.

Previous data have shown a role for STAT3 in the induction of the AP-1 factor cFos [17], and we show here that the effect of *PRKD3* silencing on STAT3 phosphorylation is concomitant with a reduction in the expression of both *FOS* and *JUN* (Fig 5A) as well as cFos protein and phosphorylated cJun (Fig 5B and 5C). We have recently reported the involvement of ATF3 in cytokine-induced *MMP13* expression [13], and assessment of *ATF3* expression following *PRKD3* silencing confirmed that it was indeed significantly reduced (Fig 6A). This was further reflected in markedly reduced nuclear ATF3 protein in cytokine-stimulated human chondrocytes following *PRKD3* gene silencing (Fig 6B and 6C).

## Discussion

**Fig 7:**
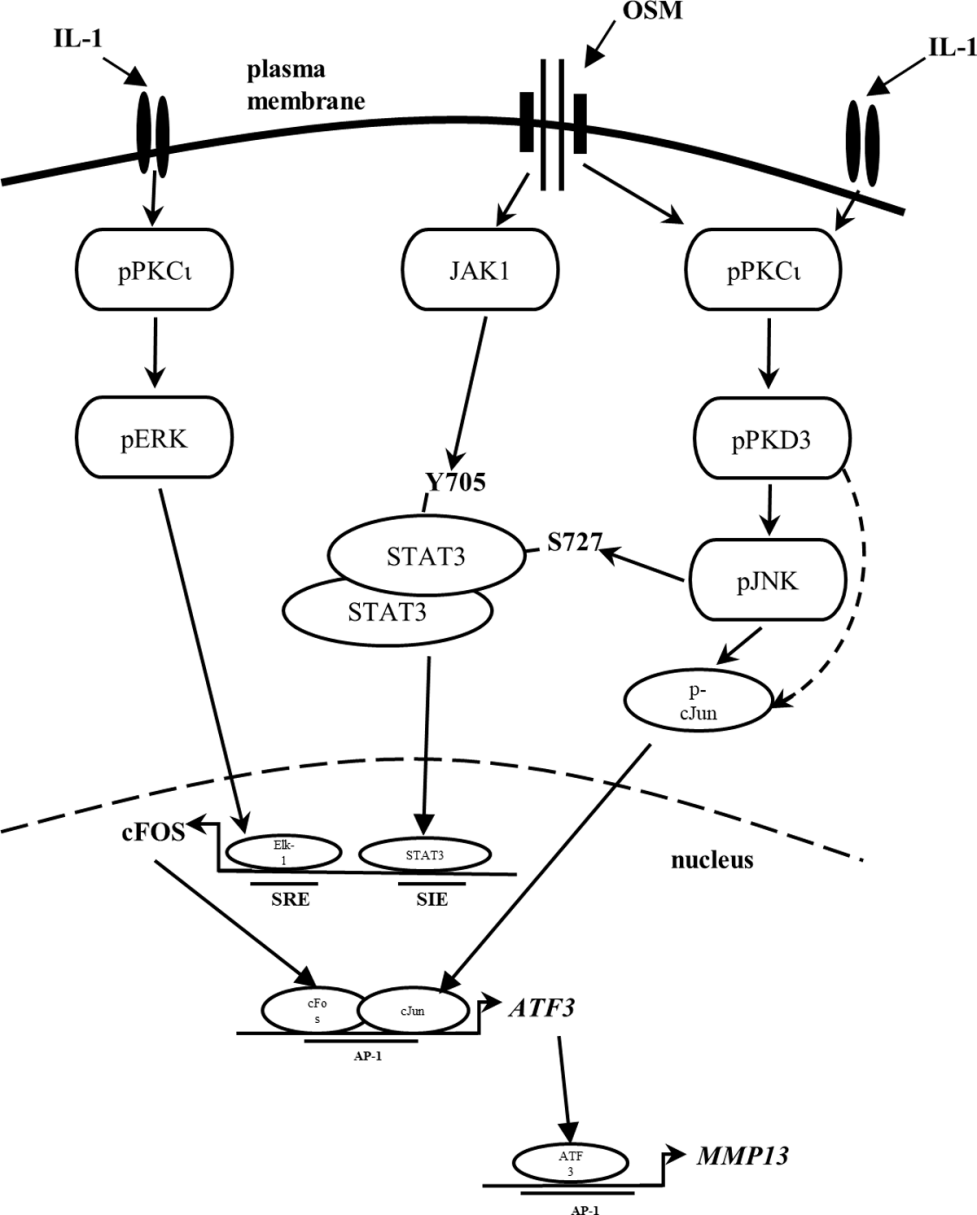
Proposed mechanism for the involvement of PKD3 in cytokine-stimulated *MMP13* induction in human chondrocytes. Stimulation by IL-1 and OSM mediates the phosphorylation and activation of PKCι, whilst OSM stimulation leads to activation of the JAK/STAT pathway including Tyr^705^ phosphorylation of STAT3. PKCι phosphorylates/activates ERK as well as PKD3 at Ser^731/735^, leading to PKD3 Ser^916^ autophosphorylation. In turn, this leads to the phosphorylation of JNK which induces Ser phosphorylation of STAT3, priming it for transcription, as well as stabilising and phosphorylating cJun (PKD3 may also directly phosphorylate cJun). Both STAT3 and ERK (via Elk1 activation) lead to the induction of *FOS*, and when combined with phospho-cJUN drives the expression of various transcriptional regulators which regulate the resulting *MMP* induction including ATF3-dependent *MMP13* expression. The scheme is based on the current study and input from Refs. (13,17–19,22,39–46).

The signal transduction associated with potent pro-inflammatory stimuli is complex although it is emerging that many of the activated pathways are common to a multitude of inflammatory mediators [13]. The interactions and cross-talk serve to markedly enhance MMP induction in human chondrocytes which ultimately drive ECM cataoblism [6,7,13,14,18,19]. We have used the catabolic stimulus of IL-1+OSM as a model inflammatory stimulus to investigate the signalling that underpins the expression of collagenolytic MMPs, in particular *MMP13* due to its high capacity to degrade the major structural component of cartilage, type II collagen [12]. Our own data and those of others [13,17,40] have shown a strong dependence on AP-1 complexes of cFos/cJun in order for *MMP* gene expression to occur, and this has been linked to AP-1 consensus motifs in the proximal promoters of both *MMP1* and *MMP13* (see [16]). Phosphorylation of PKC isoforms in human chondrocytes occurs following stimulation with IL-1 or OSM, whilst their silencing has been shown to reduce both *MMP1* and *MMP13* expression as well as the induction of *FOS* [19]. These stimuli also promote phosphorylation of the downstream PKC substrate, PKD [19]. Herein we extend these observations through the identification of PKCι as the atypical PKC isoform that mediates downstream PKD phosphorylation. Moreover, pharmacological inhibition of PKD species significantly reduced IL-1+OSM-induced *MMP1* and *MMP13* expression. However, the PKD inhibitor kb NB142-70 does not discriminate between PKD isoforms, and indeed many early studies on PKD reported findings relating to PKD as a single ‘entity’ rather than considering the three individual protein kinase isoforms. In this context, only silencing of PKD3 (*PRKD3*) mimicked the inhibition of PKDi on *MMP1/13* expression thus highlighting its role in pro-inflammatory signalling in chondrocytes. Moreover, these data also indicate that stimulation of two specific cytokine receptor pathways (IL-1 and OSM, respectively) converges on PKD3 activation via PKCι (Fig 7).

Earlier findings indicated that STAT3 is a potent mediator of cytokine-induced *MMP* expression in chondrocytes [17,19]. Evidence is provided here too indicate that *PRKD3* knockdown also modulates the activation of STATs 1 and 3 via reducing Ser^727^ phosphorylation, a requirement for maximal transcriptional activation of target genes [37]. Notably, STAT1/3 tyrosine phosphorylation was unaffected and, since nuclear import of STAT3 is independent of Tyr^705^ phosphorylation [38], the reduced nuclear STAT3 was presumably due to a PKD3-dependent alteration in the importin-α/importin-β1-Ran pathway [38]. Furthermore, the catalytic activity of PKD3 has been shown to regulate its own nuclear import through auto-phosphorylation and/or interaction with other proteins [41], such that *PRKD3* depletion prior to stimulation could markedly restrict subsequent nuclear trafficking to limit STAT3-dependent gene transcription. Indeed, *PRKD3* silencing led to a reduction in the expression of both cFOS and cJUN at both the mRNA and protein levels, as previously shown to be critical for *MMP1* and *MMP13* expression [19]; the induction of *FOS* has already been attributed to STAT3 activation [19]. Moreover, phosphorylation of cJun by JNK (Ser^63/93^) as well as by PKD isotypes (Ser^58^) has been reported [42], thus generating the transcriptionally active AP-1 (cFos/cJun) complexes associated with *MMP1/13* transcription [16]. The bone abnormalities in a gene-trap *prkd3* deletion mouse [34] also support a key role for PKD3 in the regulation of *MMP1/13* expression in chondrocytes and cartilage.

Of the other collagenolytic MMPs, *MMP14* does not possess a promoter-proximal AP-1 element [43,44] and was unaffected following *PRKD3* silencing as we would predict. Bioinformatics has identified a potential proximal AP-1 for *MMP8* [44] and, in line with our findings of reduced AP-1 expression, this MMP was also supressed. However, although many non-canonical AP-1 sequences have been shown to be functional, other reports do not support the presence of a proximal AP-1 element for *MMP8* [43,45] which would indicate other regulatory mechanisms being impacted by PKD3 activity following cytokine stimulation.

We provide further evidence for a critical role for cFos in the cytokine-mediated upregulation of *MMP1* and *MMP13* since the effect of *PRKD3* silencing on *FOS* expression was specific. Importantly, silencing of either *PRKD1* or *PRKD2* had no modulatory effects on *MMP1/13*, *FOS* or *JUN* (S4 and S6 Figs).

Despite a demonstrable role for the immediate early gene *FOS* and AP-1(cFos/cJun)-dependent induction of *MMP1* and *MMP13* (reviewed in [46]), we have also reported an absence of cFos binding to the *MMP13* promoter indicative of an indirect role for such cFos/cJun complexes [13]. Indeed, cytokine-induced *MMP13* expression involved the bZIP transcriptional regulator ATF3, the expression of which was AP-1(cFos/cJun)-dependent [13]. The obvious corollary therefore was that *ATF3* expression would be supressed following *PRKD3* silencing thus reducing its ability to transcriptionally regulate *MMP13*. Our findings herein confirm this mechanism and further highlight that although AP-1 (cFos/cJun) complexes are critical for cytokine-induced expression of genes such as *MMP13*, their role is indeed indirect: this is also most likely the case for *MMP1* [13] although no such regulatory factors have yet been reported. Furthermore, earlier findings indicate that the magnitude of *FOS* expression following pro-inflammatory stimulation appears to directly influence the subsequent levels of *MMP* expression [17] which requires the expression of AP-1-dependent transcriptional regulators [13]. This is probably the case for other examples of complex pro-inflammatory stimuli that markedly enhance *MMP* expression (eg. [5,6,8,14,17,18]. For cytokine-induced *MMP13* we further confirm the involvement of ATF3, the expression of which is critically reliant upon phosphorylation of PKD3.

## Conclusion

Our data indicate that PKD3 functions downstream of PKCι to affect the cytokine-mediated induction of *MMP1* and *MMP13* in human chondrocytes. Thus, PKD3 may represent a novel therapeutic target for consideration in the better management of inflammatory joint diseases, as well as other pathologies associated with aberrant collagenase expression and ECM catabolism.

## Supporting information

S1 Fig Confirmation of antibody specificity using human chondrocyte lysates. The specificity of each antibody used in the study was confirmed using whole cell lysates, except for MEK2 and lamin A/C (cytoplasmic and nuclear extracts, respectively), prepared as described in the Methods from primary human articular chondrocytes either unstimulated or stimulated with IL-1 (0.2 ng/ml) ± OSM (10 ng/ml). Following SDS-PAGE, proteins were transferred to PVDF membranes and probed with the indicated antibodies. Full-length blots are presented to highlight the specific immuno-reactivity of each antibody (the arrow indicates the expected molecular mass).
(PDF)

S2 Fig Effect of PKD inhibition on collagenase expression in human chondrocytes. Primary human articular chondrocytes were stimulated with IL-1 (0.2 ng/ml) alone, OSM (10 ng/ml) alone or IL-1+OSM as detailed in the Methods for 20 min. Cells were pre-treated with Gӧ6983 (20 μM; PKCi) or a DMSO vehicle control for 1 h prior to stimulation, and then lysed and immunoblotted with the indicated antibodies.
(PDF)

S3 Fig Confirmation of kinase isoform knockdown following gene silencing in primary human chondrocytes. Specificity of shRNA and siRNAs was assessed in primary human articular chondrocytes using immunoblotting (using the antibodies indicated), whilst fluorescent microscopy was also used to assess lentiviral transduction. Following transfection with siRNA specific to *PRKCI*, *PRKCZ*, *PRKD2*, *PRKD3*, *JNK1*, *JNK2* or non-targeting siCon (100 nM) (A-C) or lentiviral shRNA (MOI = 30) specific to *PRKD1* or shCon (B), as described in the Methods, cells were lysed and immunoblotted with the indicated antibodies. Fluorescent microscopy was used to assess lentiviral transduction (B). All data are representative of at least three separate chondrocyte populations.
(PDF)

S4 Fig Gene silencing of *PRKD1* or *PRKD2* does not supress cytokine-induced *MMP1/13* expression. Following transduction with lentiviral shRNA (MOI = 30) specific to *PRKD1* or shCon (A), or transfection with siRNA specific for *PRKD2* or a non-targeting siCon (100 nM) (B), primary human articular chondrocytes were stimulated with IL-1+OSM as described in the Methods for 24 h. Cell lysates were then subjected to real-time PCR (n = 6; mean ± S.E.) for *MMP1* or *MMP13* (upper and lower panels, respectively) as described in Methods. Data are presented as relative expression levels normalised to *18S rRNA* housekeeping gene, where ***, p≤0.001, **, p≤0.01 versus the relevant Control (ns = not significant). All data are representative of at least three separate chondrocyte populations.
(PDF)

S5 Fig Gene silencing of *PRKD3* selectively supresses cytokine-induced *MMP* expression. Following transfection with siRNA specific for *PRKD3* or a non-targeting siCon (100 nM), primary human articular chondrocytes were stimulated with IL-1+OSM as described in the Methods for 24 h. Cell lysates were then subjected to real-time PCR (n = 6; mean ± S.E.) for *MMP8* and *MMP14* (upper and lower panels, respectively) as described in Methods. Data are presented as relative expression levels normalised to *18S rRNA* housekeeping gene, where **, p≤0.01, versus siCon (ns = not significant). All data are representative of at least three separate chondrocyte populations.
(PDF)

S6 Fig Gene silencing of *PRKD1* or *PRKD2* does not supress cytokine-induced *FOS* and *JUN* expression. Following transduction with lentiviral shRNA (MOI = 30) specific to *PRKD1* or shCon (A), or transfection with siRNA specific for *PRKD2* or a non-targeting siCon (100 nM) (B), primary human articular chondrocytes were stimulated with IL-1+OSM as described in the Methods for 1 h. Cell lysates were then subjected to real-time PCR (n = 6; mean ± S.E.) for *FOS* or *JUN* (upper and lower panels, respectively) as described in Methods. Data are presented as relative expression levels normalised to *18S rRNA* housekeeping gene, where ns = not significant versus the relevant Control. All data are representative of at least three separate chondrocyte populations.
(PDF)
