# Supplementary material for: Protein kinase D3 modulates MMP1 and MMP13 expression in human chondrocytes
Source: PLoS One. 2018 Apr 13;13(4):e0195864. doi: 10.1371/journal.pone.0195864 (PMC5898748; doi:10.1371/journal.pone.0195864)
Supplement: S1 Fig — The specificity of each antibody used in the study was confirmed using whole cell lysates, except for MEK2 and lamin A/C (cytoplasmic and nuclear extracts, respectively), prepared as described in the Methods from primary human articular chondrocytes either unstimulated or stimulated with IL-1 (0.2 ng/ml) ± OSM (10 ng/ml). Following SDS-PAGE, proteins were transferred to PVDF membranes and probed with the indicated antibodies. Full-length blots are presented to highlight the specific immuno-reactivity of each antibody (the arrow indicates the expected molecular mass). (PDF) [file pone.0195864.s001.pdf]

S1 Fig

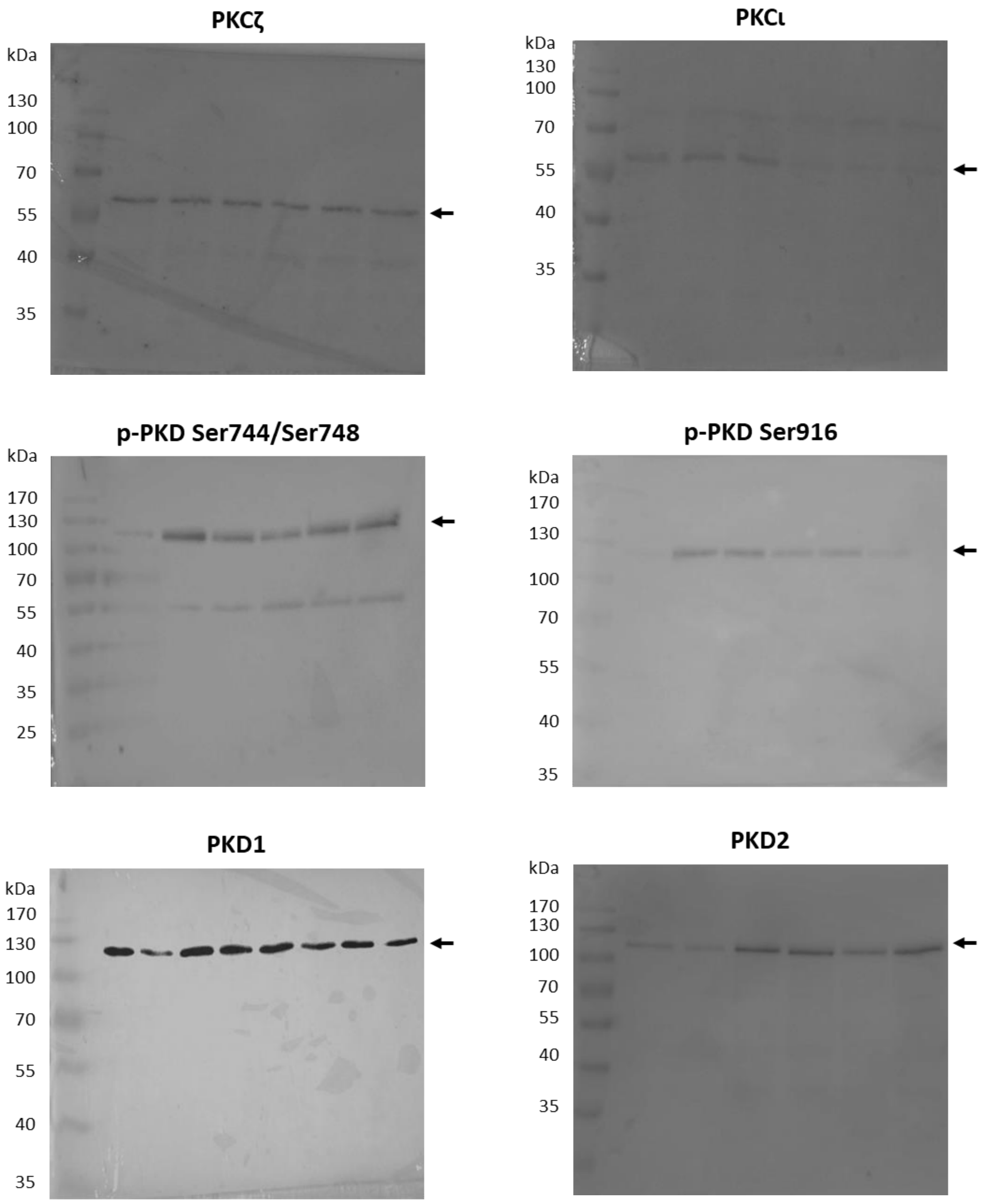
PKCζ
kDa
130
100
70
55
40
35
PKCι
kDa
130
100
70
55
40
35
p-PKD Ser744/Ser748
kDa
170
130
100
70
55
40
35
25
p-PKD Ser916
kDa
170
130
100
70
55
40
35
PKD1
kDa
170
130
100
70
55
40
35
PKD2
kDa
170
130
100
70
55
40
35

S1 Fig

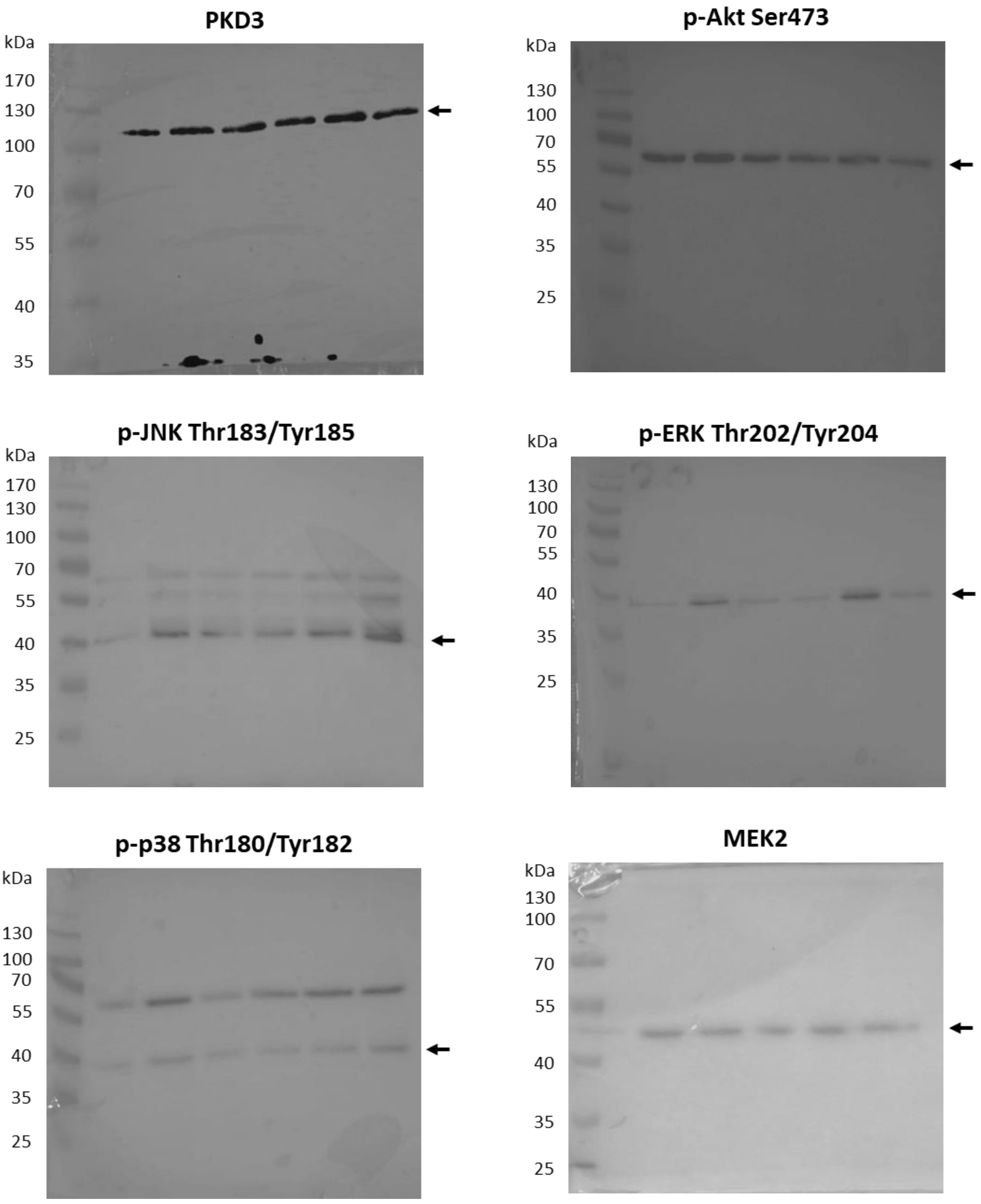
PKD3
kDa
170
130
100
70
55
40
35
p-Akt Ser473
kDa
130
100
70
55
40
35
25
p-JNK Thr183/Tyr185
kDa
170
130
100
70
55
40
35
25
p-ERK Thr202/Tyr204
kDa
130
100
70
55
40
35
25
p-p38 Thr180/Tyr182
kDa
130
100
70
55
40
35
25
MEK2
kDa
130
100
70
55
40
35
25

S1 Fig

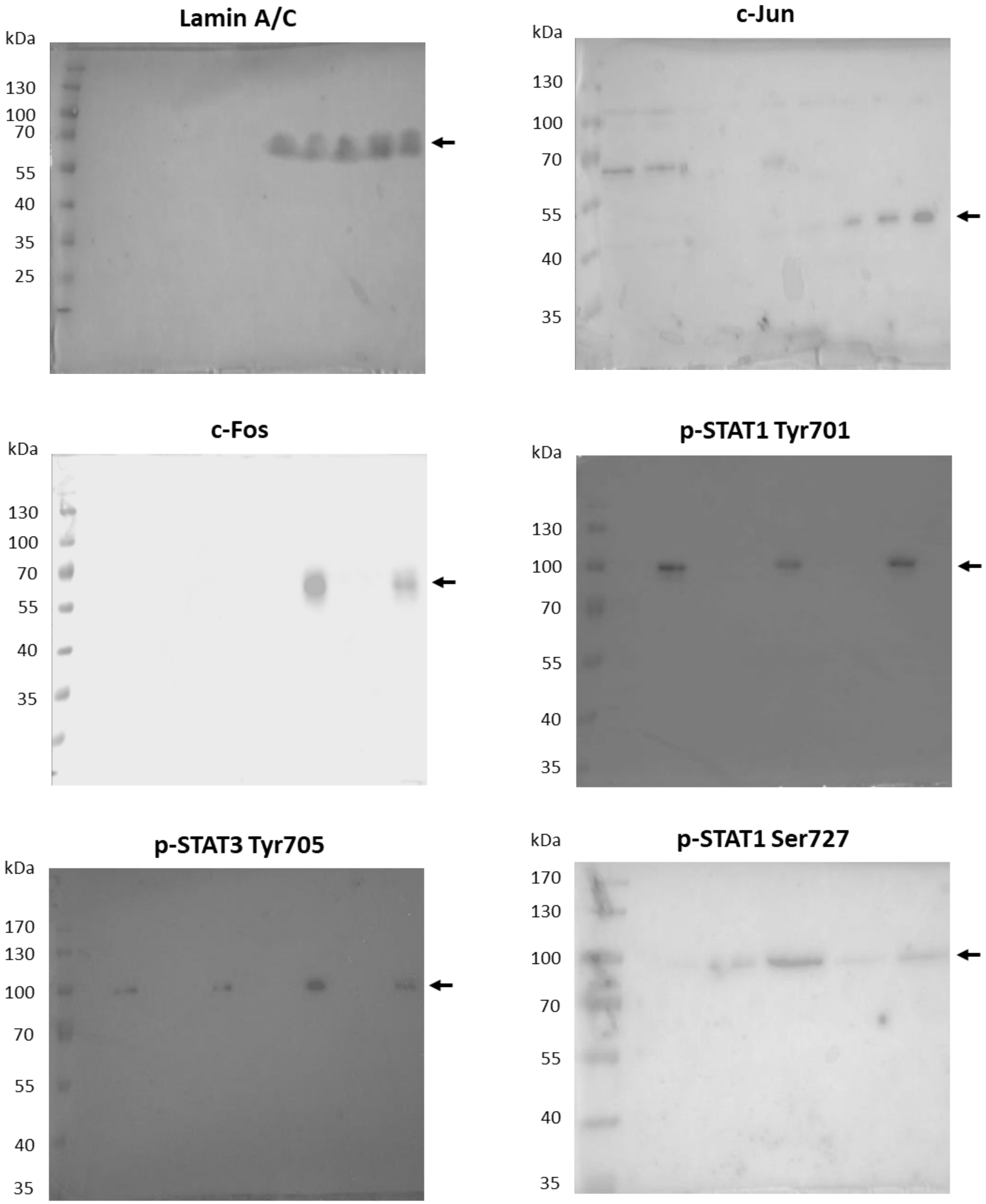

Lamin A/C
kDa
130
100
70
55
40
35
25
c-Jun
kDa
130
100
70
55
40
35
c-Fos
kDa
130
100
70
55
40
35
p-STAT1 Tyr701
kDa
130
100
70
55
40
35
p-STAT3 Tyr705
kDa
170
130
100
70
55
40
35
p-STAT1 Ser727
kDa
170
130
100
70
55
40
35

S1 Fig

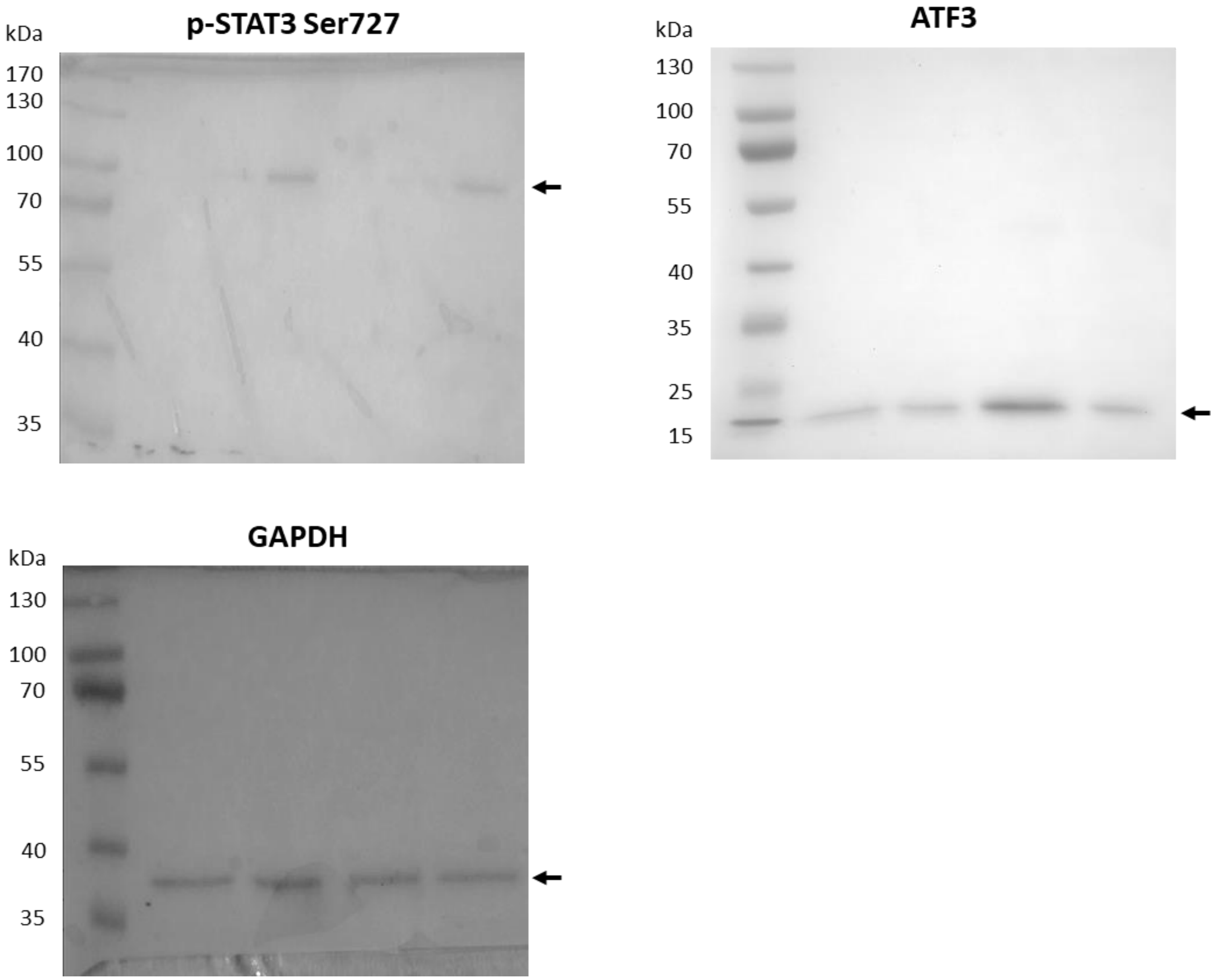
p-STAT3 Ser727
kDa
170
130
100
70
55
40
35
ATF3
kDa
130
100
70
55
40
35
25
15
GAPDH
kDa
130
100
70
55
40
35
