# Supplementary figures and images for: Protein kinase D3 modulates MMP1 and MMP13 expression in human chondrocytes
Source: PLoS One. 2018 Apr 13;13(4):e0195864. doi: 10.1371/journal.pone.0195864 (PMC5898748; doi:10.1371/journal.pone.0195864)

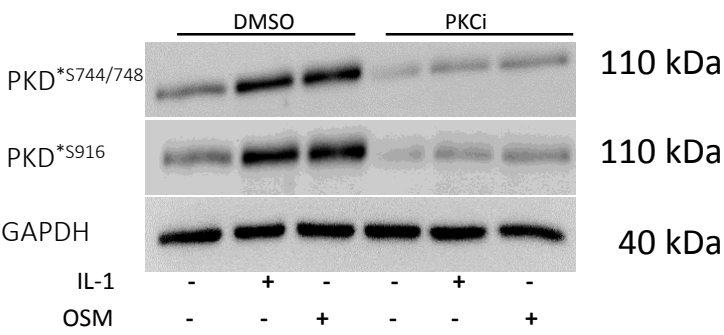

Supplement: S2 Fig — Primary human articular chondrocytes were stimulated with IL-1 (0.2 ng/ml) alone, OSM (10 ng/ml) alone or IL-1+OSM as detailed in the Methods for 20 min. Cells were pre-treated with Gӧ6983 (20 μM; PKCi) or a DMSO vehicle control for 1 h prior to stimulation, and then lysed and immunoblotted with the indicated antibodies. (PDF) [file pone.0195864.s002.pdf]

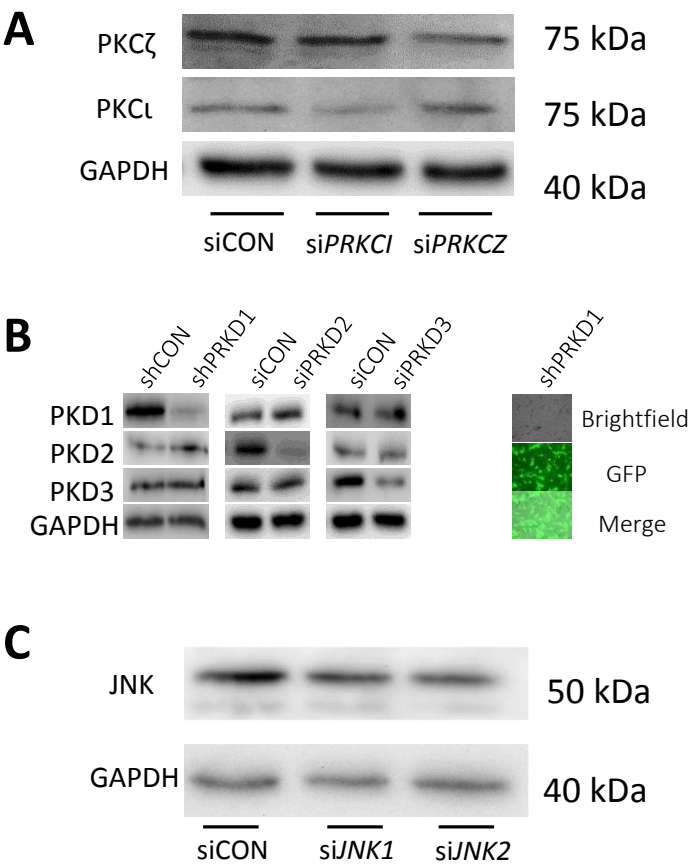

Supplement: S3 Fig — Specificity of shRNA and siRNAs was assessed in primary human articular chondrocytes using immunoblotting (using the antibodies indicated), whilst fluorescent microscopy was also used to assess lentiviral transduction. Following transfection with siRNA specific to PRKCI, PRKCZ, PRKD2, PRKD3, JNK1, JNK2 or non-targeting siCon (100 nM) (A-C) or lentiviral shRNA (MOI = 30) specific to PRKD1 or shCon (B), as described in the Methods, cells were lysed and immunoblotted with the indicated antibodies. Fluorescent microscopy was used to assess lentiviral transduction (B). All data are representative of at least three separate chondrocyte populations. (PDF) [file pone.0195864.s003.pdf]

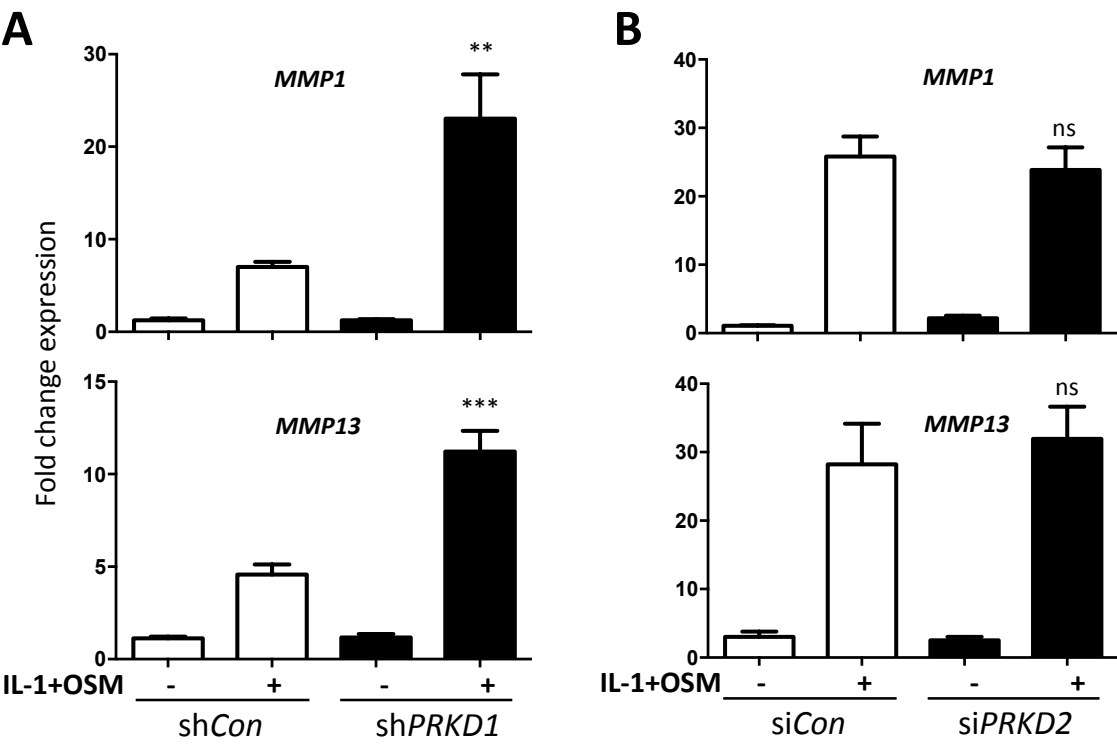

Supplement: S4 Fig — Following transduction with lentiviral shRNA (MOI = 30) specific to PRKD1 or shCon (A), or transfection with siRNA specific for PRKD2 or a non-targeting siCon (100 nM) (B), primary human articular chondrocytes were stimulated with IL-1+OSM as described in the Methods for 24 h. Cell lysates were then subjected to real-time PCR (n = 6; mean ± S.E.) for MMP1 or MMP13 (upper and lower panels, respectively) as described in Methods. Data are presented as relative expression levels normalised to 18S rRNA housekeeping gene, where ***, p≤0.001, **, p≤0.01 versus the relevant Control (ns = not significant). All data are representative of at least three separate chondrocyte populations. (PDF) [file pone.0195864.s004.pdf]

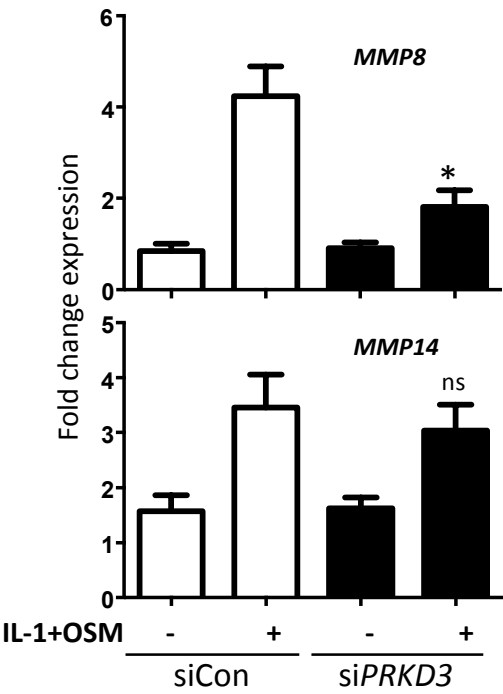

Supplement: S5 Fig — Following transfection with siRNA specific for PRKD3 or a non-targeting siCon (100 nM), primary human articular chondrocytes were stimulated with IL-1+OSM as described in the Methods for 24 h. Cell lysates were then subjected to real-time PCR (n = 6; mean ± S.E.) for MMP8 and MMP14 (upper and lower panels, respectively) as described in Methods. Data are presented as relative expression levels normalised to 18S rRNA housekeeping gene, where **, p≤0.01, versus siCon (ns = not significant). All data are representative of at least three separate chondrocyte populations. (PDF) [file pone.0195864.s005.pdf]

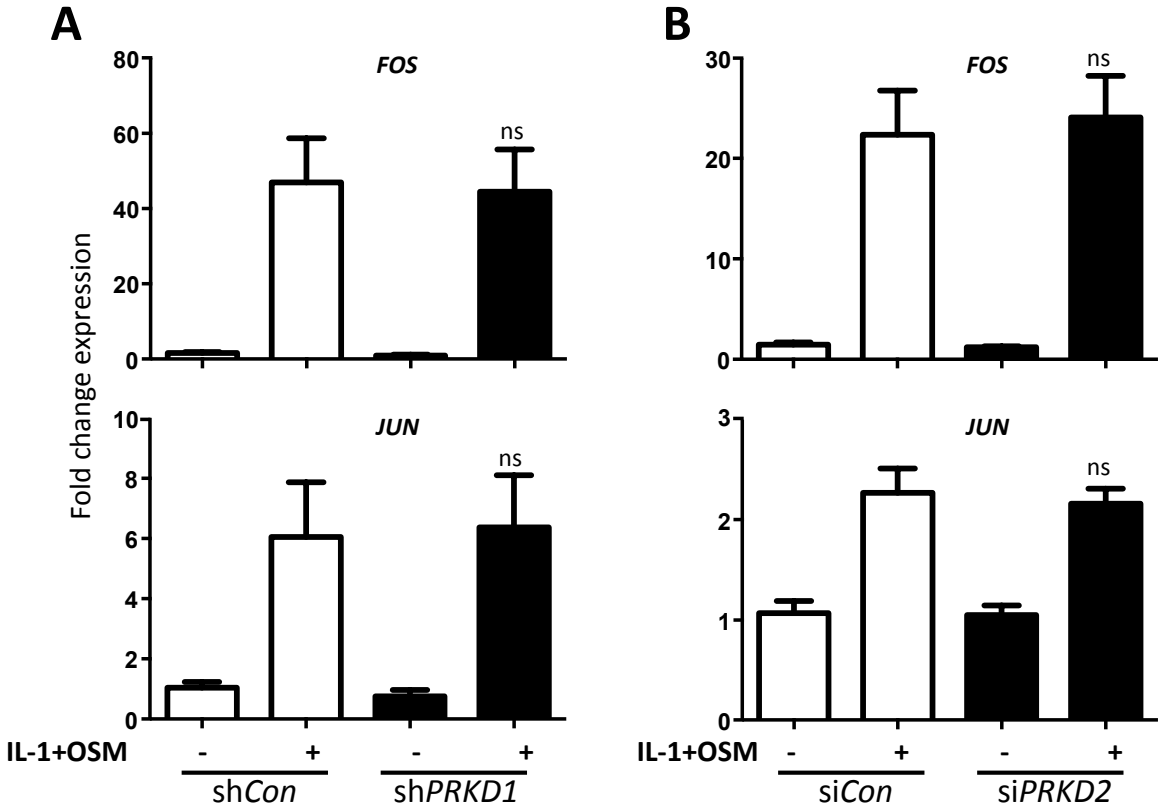

Supplement: S6 Fig — Following transduction with lentiviral shRNA (MOI = 30) specific to PRKD1 or shCon (A), or transfection with siRNA specific for PRKD2 or a non-targeting siCon (100 nM) (B), primary human articular chondrocytes were stimulated with IL-1+OSM as described in the Methods for 1 h. Cell lysates were then subjected to real-time PCR (n = 6; mean ± S.E.) for FOS or JUN (upper and lower panels, respectively) as described in Methods. Data are presented as relative expression levels normalised to 18S rRNA housekeeping gene, where ns = not significant versus the relevant Control. All data are representative of at least three separate chondrocyte populations. (PDF) [file pone.0195864.s006.pdf]
